# A Potent (R)-alpha-bis-lipoyl Derivative Containing 8-Hydroxyquinoline Scaffold: Synthesis and Biological Evaluation of Its Neuroprotective Capabilities in SH-SY5Y Human Neuroblastoma Cells

**DOI:** 10.3390/ph6010054

**Published:** 2013-01-07

**Authors:** Ivana Cacciatore, Erika Fornasari, Leonardo Baldassarre, Catia Cornacchia, Stefania Fulle, Ester Sara Di Filippo, Tiziana Pietrangelo, Francesco Pinnen

**Affiliations:** 1 Dipartimento di Farmacia, Università degli Studi “G. D’Annunzio”, Via dei Vestini 31, 66100 Chieti, Italy; 2 Dipartimento di Neuroscienze e Imaging; IIM - Istituto Interuniversitario di Miologia Università degli Studi “G. D’Annunzio”, Via dei Vestini 31, 66100 Chieti, Italy; 3 Ce.S.I. Centro Studi Invecchiamento, Fondazione d’Annunzio, Via Colle dell’Ara, 66100 Chieti, Italy

**Keywords:** (R)-alpha-lipoic acid, 8-hydroxyquinoline, neuroprotective agent, antioxidants, neurodegenerative diseases.

## Abstract

A novel bis-lipoyl derivative containing 8-hydroxyquinoline scaffold (LA-HQ-LA, **5**) was synthesized as a new multifunctional drug candidate with antioxidant, chelant, and neuroprotective properties for the treatment of neurodegenerative diseases. We have investigated the potential effectiveness of LA-HQ-LA against the cytotoxicity induced by 6-OHDA and H_2_O_2_ on human neuroblastoma SH-SY5Y cell line. Our outcomes showed that LA-HQ-LA resulted in significant neuroprotective and antioxidant effects against H_2_O_2_- and 6-OHDA-induced neurotoxicity in human neuroblastoma SH-SY5Y cells, as assessed by MTT assay. In particular, it showed potent neuroprotective effects against 6-OHDA in RA/PMA differentiated cells at all the tested concentrations.

## 1. Introduction

Neurodegenerative diseases comprise a condition in which nerve cells from brain and spinal cord are lost leading to either functional loss (ataxia) or sensory dysfunction (dementia). Mitochondrial dysfunctions, excitotoxicity, and, finally, apoptosis have been reported as pathological causes for aging and neurodegenerative diseases such as Parkinson’s disease (PD), Alzheimer’s disease (AD), multiple sclerosis (MS), and amyotrophic lateral sclerosis (ALS) [1]. Although any disease mentioned above is characterized by its own molecular mechanism and clinical onset, common pathways can be identified as: 1) oxidative stress and free radicals formation; 2) metal dyshomeostasis; 3) protein misfolding and aggregation; and 4) mitochondrial dysfunctions [2]. Oxidative stress plays a key role in neurodegenerative diseases: it arises due to an imbalance between pro-oxidant/antioxidant homeostasis that further takes part in generation of ROS and free radicals potentially toxic for neuronal cells [3]. Furthermore, in all forms of ROS generation molecular oxygen needs to be activated by a range of metallo-enzymes, thus facilitating ROS generation upon interaction of redox metals with O_2_ using various catalytic pathways. Since free radicals are toxic to cells, under normal circumstances, cells have an efficient regulating system for O_2_ and metal ion interaction leading to free radicals and ROS generation [4]. In fact, during the Fenton reaction, hydroxyl radicals are produced from hydrogen peroxide in the presence of a metal in a low oxidation state:

H_2_O_2_ + M^n+^ → HO^-^ + HO∙ + M^(n+1)+^

Fenton chemistry may occur in neurons of the nervous tissue where levels of both hydrogen peroxide and cerebral biometals—such as Fe^3+^; Cu^2+^; and Zn^2+^—are found. Several studies have indicated that cerebral biometal dyshomeostasis and oxidative stress are intimately associated [5]. An *in vitro* AD model has shown that both Aβ-40 and Aβ-42 deposits are formed after incubation of immobilized β-amyloid oligomers with Cu^2+^, Zn^2+^, or Fe^3+^. In these conditions, Fe^3+^ promoted the deposition of fibrillar amyloid plaques, while Cu^2+^ and Zn^2+^ only induced the formation of amorphous aggregates [5]. In an *in vitro* PD model, it has been found that Fe^3+^ enhanced intracellular aggregation of α-synuclein and led to the formation of advanced glycation end products. The accumulation of these factors strongly contributed to the progression of the neurodegenerative process [6].

Increasingly medicinal chemistry approaches are currently under study to discover new drugs able to remove excess of specific metals [7,8] and to prevent or block the oxidative process that characterizes PD and AD [9,10,11,12]. Taking into account that drugs with two or more useful biological activities for the same pathology may represent an important pharmacological advance, we are currently interested in multifunctional drugs that combine potent antioxidant, chelant, and neuroprotective properties in a single molecule for the treatment of PD and AD [13,14,15,16]. For this purpose, to design a novel class of compounds with a multimodal mechanism of action, we selected the hydroxyquinoline (HQ) scaffold as a privileged structure since it is a clinically relevant bioactive metal chelator. Recently, 8-hydroxyquinoline (8-HQ, **1**) derivatives have found application in PD and AD drug discovery [17] since 8-HQ: 1) is able to cross the blood–brain barrier (BBB) [18]; 2) is a strong iron chelator with antioxidant property [19,20,21]; and 3) is able to protect against the precipitation of β-amyloid plaques in presence of Cu^2+^, Fe^3+^, Zn^2+^—compared to clioquinol—due to its ability to chelate these metals [22].

The aim of this work was to combine the antioxidant and neuroprotective properties of (R)-alpha-lipoic acid (LA, **3**) and the chelant activities of 8-HQ (**1**) [23] to obtain a novel multi-target ligand, LA-HQ-LA (**5**) with multifunctional neuroprotective profile. LA-HQ-LA was obtained by linking via two ester bonds the 8-HQ derivative (5-hydroxymethyl-8-hydroxyquinoline, **2**) to LA, thus increasing the lipophilicity of this molecule. LA-HQ-LA can cross plasma membranes and release HQ and two molecules of LA, thus triggering a significant decrease in oxidative stress from human SH-SY5Y neuroblastoma cells. In addition, due to the different chemical nature of the ester bonds, the derivative **5** could gradually provide a continuative and time-controlled release of LA—an elevator of GSH levels that are lower in some cerebral areas of patients affected by neurodegenerative diseases [24]—and HQ directly to specific groups of neurons characterized by cellular stress and metals accumulation.

## 2. Results and Discussion

**Scheme 1:**
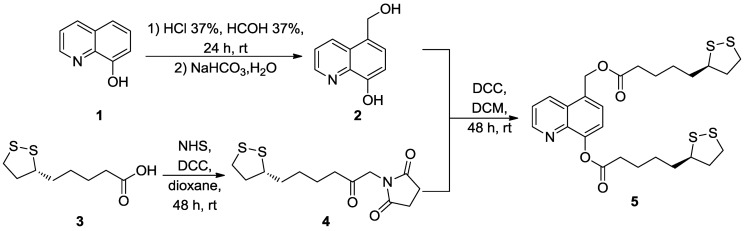
Synthesis of LA-HQ-LA (**5**).

Starting from 8-HQ (**1**), the required starting material 5-hydroxymethyl-8-hydroxyquinoline (**2**) was obtained in good yield, using a known procedure [25]. The new multi-target ligand LA-HQ-LA (**5**) was synthesized by direct condensation of 5-hydroxymethyl-8-hydroxyquinoline (**2**) and LA-NHS (**4**), previously prepared as reported by Nefkens *et al.* [26] (Scheme 1). The chemical structure of LA-HQ-LA was confirmed by ^1^H-,^13^C-NMR, IR, and MS spectra data.

The neuroprotective and antioxidant capacities of LA-HQ-LA against oxidative stress were assayed by using the human SH-SY5Y neuroblastoma cell line, which is a reliable model for studying the neurotoxic effect of agents such as H_2_O_2_, 6-OHDA, and frequently used for elucidating the mechanisms of neurodegenerative diseases [27].

**Figure 1:**
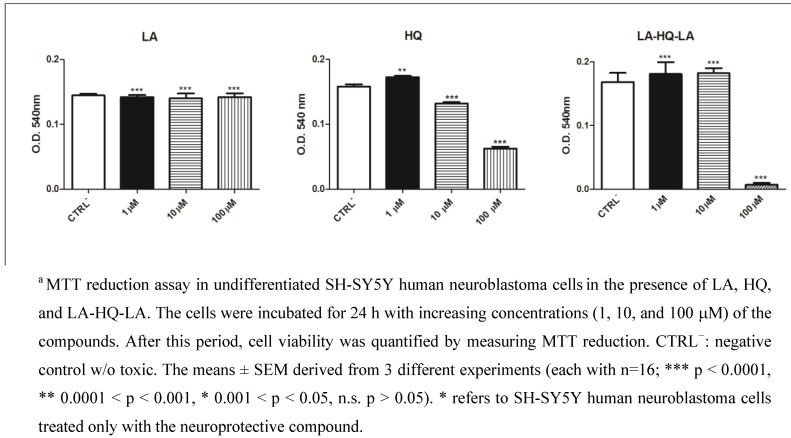
Dose-response effects of LA, HQ, and LA-HQ-LA in undifferentiated SH-SY5Y human neuroblastoma cells^a^.

**Figure 2:**
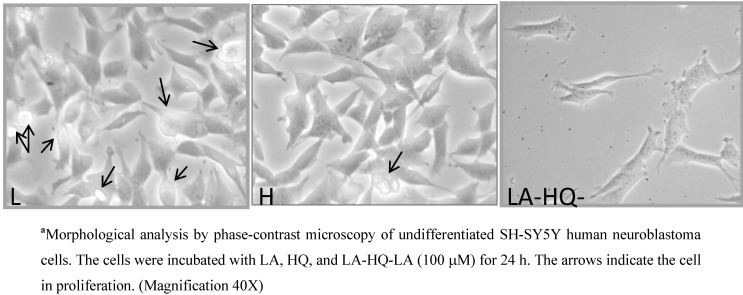
Morphological analysis of undifferentiated SH-SY5Y human neuroblastoma cellsin the presence of LA, HQ, and LA-HQ-LA.^a^

**Figure 3:**
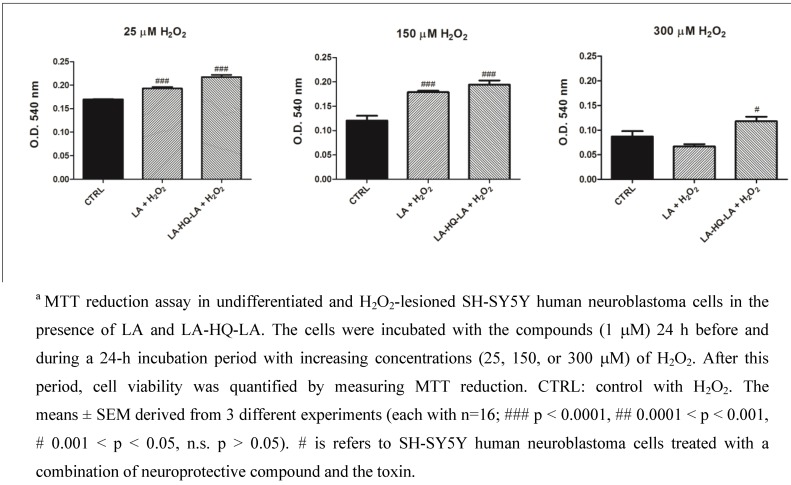
Neuroprotective effects of LA and LA-HQ-LA in undifferentiated and H_2_O_2_-lesioned SH-SY5Y human neuroblastoma cells.^a^

First of all, to define the suitable concentration range, the effects on cell proliferation of LA, HQ, and LA-HQ-LA were determined by colorimetric MTT assay (Figure 1). Thus, we performed dose-response experiments (with compound concentrations of 1, 10, and 100 μM) to verify if, 24 h after the treatment, the compounds added to the cells had any effect on the cell proliferative capacity. The compound concentrations of 1 and 10 μM did not show significant differences compared to the control, while at 100 μΜ, an antiproliferative activity was observed (Figure 1, Figure 2 and Figure 3). In particular, at 100 μM, LA still retained a proliferative activity, while LA-HQ-LA resulted in an antiproliferative activity, since there are no visible proliferating cells (Figure 2). Based on the results obtained, the utilized compound concentration in all the experiments reported in this study was 1 μM.

**Figure 4:**
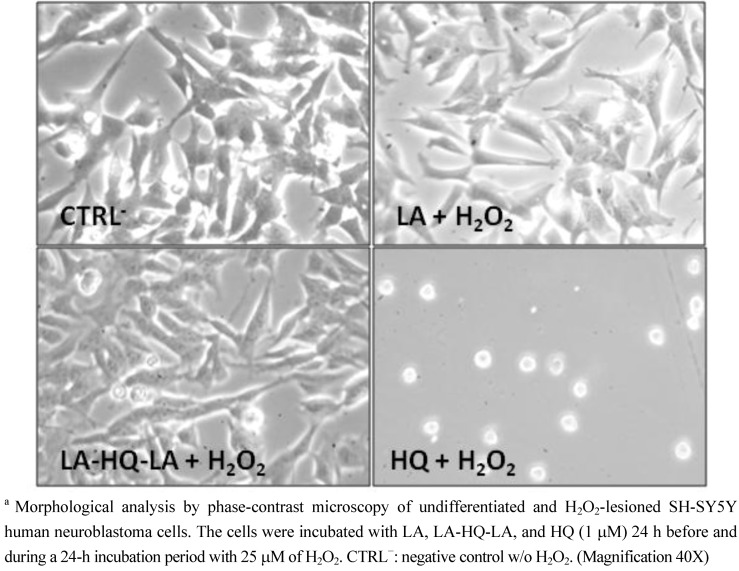
Morphological analysis of undifferentiated and H_2_O_2_-lesioned SH-SY5Y human neuroblastoma cells in the presence of LA, LA-HQ-LA, and HQ.^a^

Because the sensitivity of cells to toxic agents may be dependent on the state of their neuronal differentiation, we used undifferentiated and RA-differentiated SH-SY5Y cells to study the neuroprotective effect of our compounds following H_2_O_2_ insult [28,29]. Thus, undifferentiated SH-SY5Y cells were pre-treated with LA, HQ, and LA-HQ-LA for 24 h and then exposed to H_2_O_2_ (25-150-300 μM) for other 24 h (cell viability was detected using MTT assay as shown in Figure 3). Both compounds (LA-HQ-LA and LA) showed a significant protective effect against H_2_O_2_ at 25 and 150 μM H_2_O_2_. Moreover, the neuroprotective effect of LA-HQ-LA was appreciably higher in respect to LA and the control at 300 μM. In Figure 3, we did not insert the results obtained for HQ since, as evidenced by the morphological evaluation (Figure 4), at 1 μM and in presence of 25 μM of H_2_O_2_ it was demonstrated as toxic for the undifferentiated SH-SY5Y cells. This deleterious effect on SH-SY5Y cells could be due to a synergic action between HQ and H_2_O_2_. HQ, being a metal chelator of iron, copper, and zinc (transition metals that react easily with ROS), avoids the Haber-Weiss reaction between the metal and the superoxide anion; in these oxidative stress conditions, superoxide anion is overproduced, thereby damaging cells. On the other hand, H_2_O_2_ directly produces a high quantity of ROS further damaging the cells. These two combined actions determine a deleterious synergic effect on cells.

**Figure 5:**
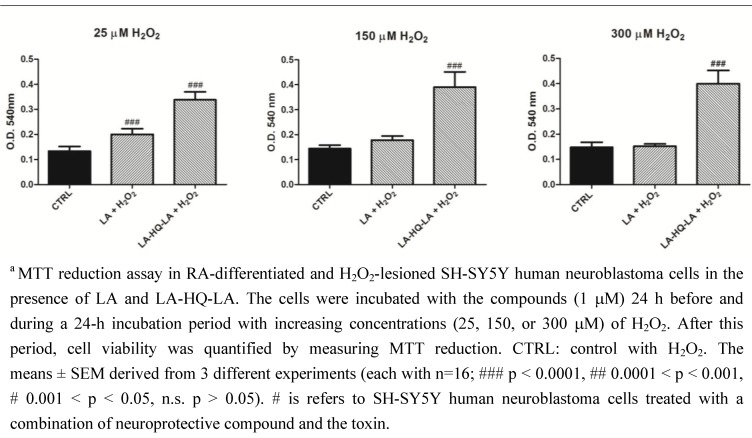
Neuroprotective effects of LA and LA-HQ-LA in RA-differentiated and H_2_O_2_-lesioned SH-SY5Y human neuroblastoma cells.^a^

Many evidences indicated that the neuronal differentiation of SH-SY5Y neuroblastoma cells seems appropriate for studying neurotoxicity since the proliferation rate is limited and cells morphologically resemble the neuronal phenotype [30]. Thus, we differentiated our cells with RA to increase the cholinergic properties of SH-SY5Y cell line and we lesioned them with increasing concentrations (25–150–300 μM) of H_2_O_2_ (Figure 5). Results confirmed a significant neuroprotective effect of LA-HQ-LA, at all doses of H_2_O_2_, against LA and control. These results confirmed several previous reports [28,31,32] which showed that the differentiation of SH-SY5Y cells by RA enhanced their resistance to the action of neurotoxic agents. In fact, comparing Figure 3 and Figure 5, indicating cellular viability, we observed that at high concentrations of H_2_O_2_ (300 μ M), the optical density O.D. values were reduced more in the undifferentiated than differentiated SH-SY5Y cells. Thus, the undifferentiated cells were much more vulnerable to H_2_O_2_ than RA-treated ones.

**Figure 6:**
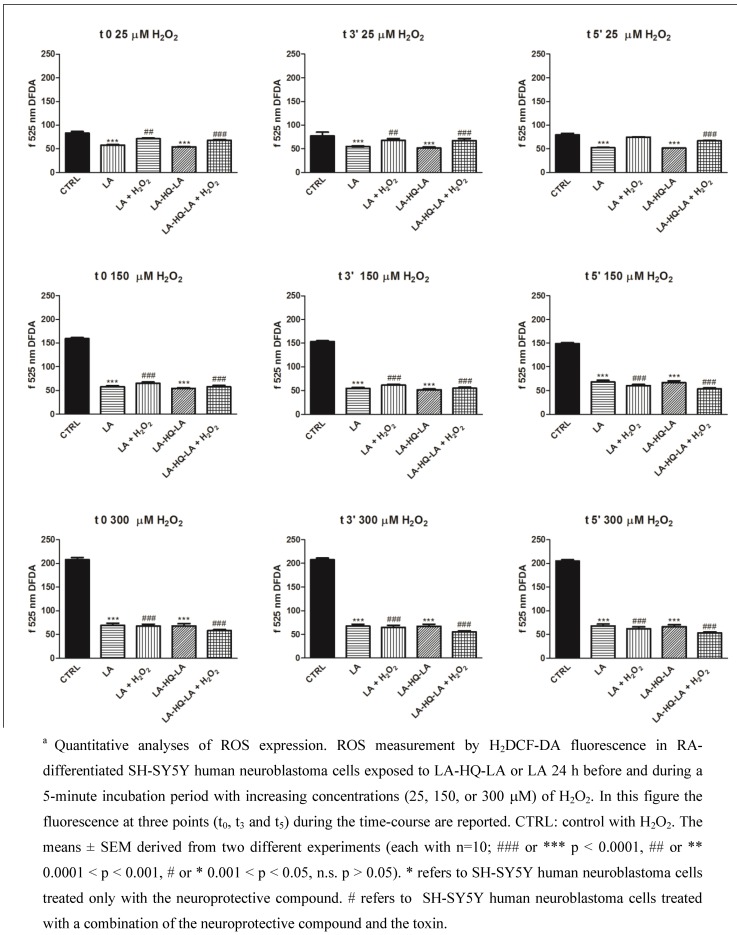
Antioxidant capabilities of LA and LA-HQ-LA in RA-differentiated and H_2_O_2_-lesioned SH-SY5Y human neuroblastoma cells ^a^.

In the above reported experiments, exposing SH-SY5Y neuroblastoma cells to increasing concentrations of H_2_O_2_, we induced reactive oxygen species (ROS) generation. An overproduction of ROS and a lower antioxidant capability of the cells result in oxidative stress that characterizes several neurodegenerative pathologies. To accurately measure ROS and the cell capability to counteract this insult, we used cell permeable fluorescent and chemiluminescent probes. 2′-7′-Dichlorodihydrofluorescein diacetate (H_2_DCF-DA) is one of the most widely used techniques for directly measuring the redox state of a cell [33]. Our results showed that, at t_0_-t_5_ and at all the concentrations of H_2_O_2_, LA-HQ-LA and LA exerted a powerful antioxidant effect returning ROS levels similar to the control (Fig. 6). These data confirmed that our compound possesses good antioxidant and neuroprotective capabilities.

Comparing Figure 3, Figure 5, and Figure 6 we can observe that LA-HQ-LA showed a long-lasting neuroprotective activity respect to LA only in long-term experiments (Fig. 3, 5) probably due to a time-controlled release of its components.

**Figure 7:**
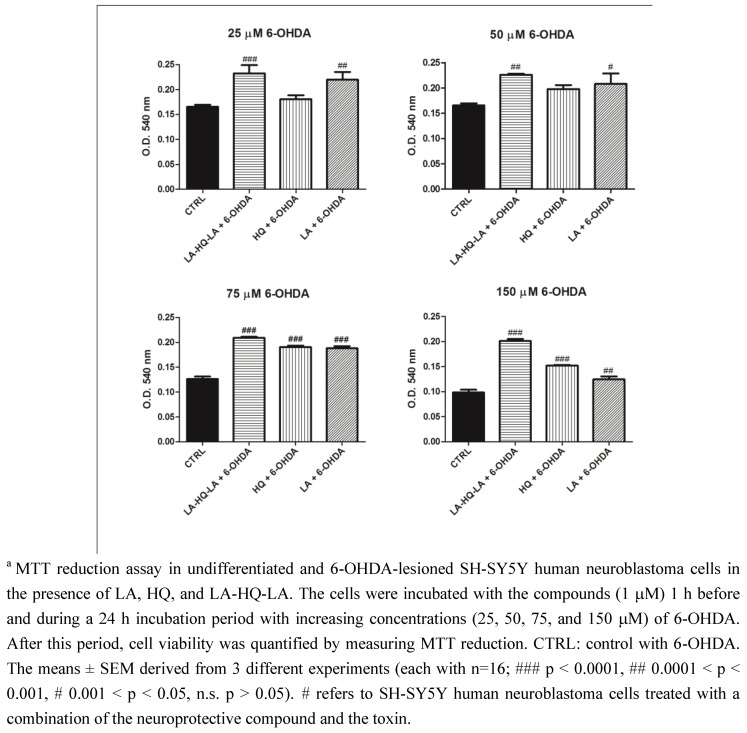
Neuroprotective effects of LA, HQ, and LA-HQ-LA in undifferentiated and 6-OHDA-lesioned SH-SY5Y human neuroblastoma cells^a^.

**Figure 8:**
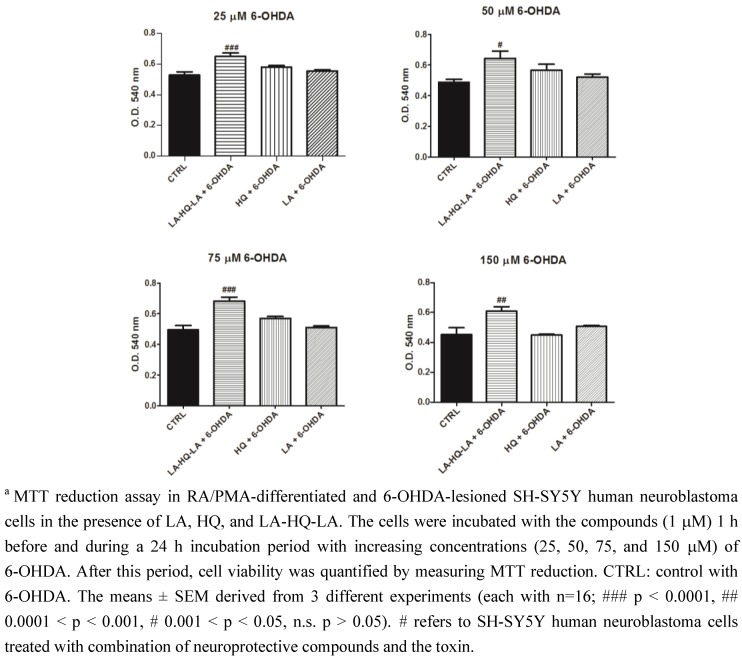
Neuroprotective effect of LA, HQ, and LA-HQ-LA in RA/PMA-differentiated and 6-OHDA-lesioned SH-SY5Y human neuroblastoma cells.^a^

To further investigate the neuroprotective role in PD, it was necessary to differentiate SH-SY5Y neuroblastoma cells toward the DAergic phenotype using RA/PMA [34,35]. We used the neurotoxin 6-OHDA as a toxic agent to lesionate the cells, as it is commonly used as a dopaminergic degeneration model for both *in vitro* and *in vivo* studies [36]. Like DA, 6-OHDA is quickly oxidized to form a variety of free radical species, and also induces ROS-dependent apoptosis in dopaminergic cells. In this second toxicity model, the neuroprotective effect of LA, HQ, and LA-HQ-LA against oxidative stress was evaluated by using the neurotoxin 6-OHDA in both the undifferentiated and RA/PMA-differentiated cells. The undifferentiated SH-SY5Y cells were treated with LA, HQ, and LA-HQ-LA for 1 h and then exposed to increasing concentrations of 6-OHDA (25-50-75-150 μM). After further 24 h of incubation, the cultures were assessed for viability by MTT assay (Figure 7). LA-HQ-LA was the most neuroprotective compound, among the ones investigated, mostly at the strongly neurotoxic 6-OHDA concentration of 150 μM. In RA/PMA-differentiated cells (Figure 8), LA-HQ-LA showed a strong neuroprotective effect at all the used concentrations of 6-OHDA. Also in these experiments, we observed a major susceptibility to toxic insult of undifferentiated respect to differentiated cells (Figure 7 and Figure 8).

Our data showed that LA-HQ-LA has a significant neuroprotective effect against both H_2_O_2_ and 6-OHDA, higher than LA and HQ, up to 150 μM. However, LA-HQ-LA showed a different behavior in the response (mainly with 6-OHDA) in differentiated cells compared to the undifferentiated ones. This difference probably depends on the variable sensitivity of cells against neurotoxic agents. In fact, RA/PMA-differentiated cells exhibit 6-fold higher levels of dopamine D_2_ and D_3_ receptors in respect to undifferentiated or RA-differentiated cells [37]. Furthermore, it is also reasonable that LA-HQ-LA showed the greatest effect against neurotoxic agents, because LA itself is a neuroprotective agent for dopaminergic neurons [38]. The obtained results confirm that LA-HQ-LA is a valid molecule, potentially more effective in respect to LA and HQ. In particular, LA-HQ-LA—at low concentrations (1 μM)—might be a good therapeutic choice in diseases such as PD, where oxidative stress caused by oxidants plays a key role.

## 3. Experimental Section

### 3.1. Chemistry

HQ (**1**) and LA (**3**) were purchased from Sigma Chemical Co. (St Louis MO, USA). All other chemicals used were of the highest commercially available purity. Chromatographic purifications were performed on silica gel using column chromatography (Merck 60, 70–230 mesh ASTM silica gel), and compounds were detected with UV light (λ = 254 nm). Optical rotations were taken at 20 °C with a Perkin-Elmer 241 polarimeter (Santa Clara, CA, USA). IR spectra were recorded with a Varian 1000 FT-IR spectrometer. NMR spectra were recorded with a Varian VXR-300 spectrometer (Varian Medical Systems, Inc., Palo Alto, CA, USA). Chemical shifts are reported in parts per million (*δ*) downfield from the internal standard tetramethylsilane (Me_4_Si). Mass spectra were obtained by electrospray ionization (ESI) in positive mode using a LCQ (Thermo Finnigan) ion trap mass spectrometer (San Jose, CA, USA) equipped with an electrospray ionization (ESI) source. The capillary temperature was set at 300 °C and the spray voltage at 4.25 kV. The fluid was nebulized using nitrogen (N_2_) as both the sheath gas and the auxiliary gas.

The identity of LA-HQ-LA was confirmed by NMR, IR, and MS spectra; the homogeneity was confirmed by TLC on silica gel Merck 60 F254. Before performing biological studies, the purity of LA-HQ-LA was checked by HPLC analysis; it was determined by analytical HPLC using a Waters 600 HPLC (Waters Co., Milford, MA, USA) equipped with an X-Bridge BEH130 C-18, 5 µm, 4.6 × 250 mm column with Waters 2996 PDA detector, and an isocratic elution using water (0.1% TFA) at flow rate of 1 mL/min. LA-HQ-LA was obtained with purity greater than 98%, determined by analytical HPLC at 280 nm.

#### 3.1.1. Synthesis of 5-Hydroxymethyl-8-hydroxyquinoline (**2**)

A mixture of **1** (2 g, 13.77 mmol), concentrated hydrochloric acid (4.4 mL), and 37% formaldehyde (2.2 mL) was treated with hydrogen chloride gas and stirred overnight. The reaction mixture was filtered and the precipitate was dried giving the 5-hydroxymethyl-8-hydroxyquinoline hydrochloride as a yellow solid (yield: 93%). The product was then solubilized with water and added with NaHCO_3_ (3.9 g, 46.48 mmol); the precipitate was dissolved and extracted with CHCl_3_/NaCl, dried over Na_2_SO_4_, and the solvent was removed under vacuum obtaining **2** as a white solid (yield: 68%). ^1^H-NMR (300 MHz, DMSO-d_6_) δ: 4.78–4.83 (2H, m, CH_2_, HQ), 5.16 (1H, s, OH, HQ), 6.96–7.57 (3H, m, Ar, HQ), 8.47–8.83 (2H, m, Ar, HQ), 9.70 (1H, s, br, OH); ^13^C-NMR (300 MHz, DMSO-d_6_): 111.35–153.07 (9 × *C*H, HQ).

#### 3.1.2. Synthesis of 1-(-5-[1,2]dithiolan-3-yl-pentanoyl)-pyrrolidine-2,5-dione (LA-NHS, **4**)

A solution of **3** (3.69 g, 19.2 mmol) in dry dioxane (15.4 mL) was slowly added to a mixture of NHS (2.3 g, 20 mmol) and DCC (4.12 g, 20 mmol) in dry dioxane (37 mL) and stirred for 48 h at room temperature. After filtration of the precipitate, the solvent was evaporated, the residue was taken up in 2-propanol, and left overnight at 4 °C. The resulting white precipitate **4** was then filtered and dried (yield: 60%). ^1^H-NMR (300 MHz, DMSO-d_6_) δ: 1.25 ( 2H, m, CH_2_, LA), 1.37–1.62 (4H, m, 2 × CH_2_, LA), 1.84 (2H, m, CH_2_, LA), 2.29 (2H, t, CH_2_, LA), 2.42–2.51 (3H, m, CH_2_ and CH, LA), 2.78 (4H, t, 2 x CH_2_, NHS); ^13^C-NMR (300 MHz, DMSO-d_6_): 28.1 (*C*H_2_, LA), 29.3 (*C*H_2_, LA), 33.0 (2 x *C*H_2_, NHS), 33.5 (*C*H_2_, LA), 34.8 (*C*H_2_, LA), 37.9 (*C*H_2_, LA), 42.9 (*C*H_2_, LA), 55.7 (*C*H, LA), 107.4 (*C*H, NHS), 172.3 (*C*O, LA), 205.2 (2 × *C*O, NHS).

#### 3.1.3. Synthesis of LA-HQ-LA (**5**)

5-Hydroxymethyl-8-hydroxyquinoline (**2**) (440 mg, 2.5 mmol) was dissolved in dry CH_2_Cl_2_ (72 mL) and then added to a stirring mixture of **4** (3.03 g, 10 mmol) and DCC (2.06 g, 10 mmol) in dry CH_2_Cl_2_ (72 mL). The reaction mixture was left at room temperature for 48 h under stirring, then the solution was filtered and the solvent was removed. The obtained residue was chromatographed using petroleum ether: AcOEt (6:4) as eluant to give LA-HQ-LA as a yellow oil (yield: 57%). *R_f_* = 0.72 (petroleum ether:AcOEt (6:4); [α]_D_^2^°= + 20.0 (*c*=1 in MeOH); ^1^H-NMR (300 MHz, CDCl_3_) δ: 1.42 (2H, m, CH_2_, LA), 1.8 (12H, m, 6 × CH_2_, LA), 2.36 (2H, m, CH_2_, LA), 2.47 (2H, m, CH_2_, LA), 2.81 (2H, t, CH_2_, LA), 3.12 (4H, m, 2 x CH_2_, LA), 3.49 (1H, m, LA), 3.63 (1H, m, LA), 5.52 (2H, s, Ar-CH_2_-O), 7.42 (1H, d, HQ), 7.50 (1H, m, HQ), 7.60 (1H, d, HQ), 8.38 (1H, d, HQ), 8.93 (1H, d, HQ). ^13^C-NMR (300 MHz, CDCl_3_) δ: 24.85 (CH_2_, LA), 24.93 (CH_2_, LA), 28.85 (OOCCH_2_-*C*H_2_, LA), 28.96 (OOCCH_2_-*C*H_2_, LA), 34.18 (OOC-*C*H_2_, LA), 34.24 (OOC-*C*H_2_, LA), 34.77 (CH_2_, LA), 34.9 (CH_2_, LA), 38.74 (2 x CH_2_, LA), 40.41 (CH_2_, LA), 40.48 (CH_2_, LA), 56.49 (CH, LA), 56.65 (CH, LA), 63.48 (COO-*C*H_2_-HQ), 121.07 (*C*H, HQ), 122.27 (*C*H, HQ), 128.25 (*C*H, HQ), 130.30 (*C*-OCO, HQ), 132.56 (*C*H, HQ), 148.34 (*C*-N, HQ), 150.56 (N-*C*H, HQ), 172.48 (*C*OO, LA), 173.39 (*C*OO, LA); IR (KBr): *v*^~^= 3409, 2932, 2283, 1733, 1461, 1376, 1259, 1182, cm^−1^; MS (ESI) *m*/*z* 552.12 (M+H)^+^.

### 3.2. Neuroprotective Studies

#### 3.2.1. SH-SY5Y Cell Culture

Human SH-SY5Y neuroblastoma cells (EGACC, Sigma–Aldrich, UK) were grown at 37 °C, in a humid 5% CO_2_, in Dulbecco’s Modified Eagle’s medium supplemented with 10% heat-inactivated fetal bovine serum, penicillin (100 U/mL), streptomycin (100 μg/mL), and 1% L-glutammine. SH-SY5Y cells were plated onto 96-well plates (2,700 cell/well). Undifferentiated cells (UN) were grown for 24 h in normal medium and then incubated with the compounds (LA, HQ, and LA-HQ-LA). After 24 h of incubation, the cultures were assessed for viability by using a colorimetric assay based on the ability of living cells to reduce a tetrazolium-based compound to a blue formazan product (MTT assay). At the end of incubation, 20 μL of MTT (5 mg/mL in PBS) were added to each well and the incubation continued for an additional 3 h at 37 °C. The plate was centrifuged at 2000 rpm for 15 min. The MTT solution was carefully decanted off and formazan crystals were dissolved with 200 μL DMSO at 37 °C for 30 min. Finally, samples were read in a Titertek Multiscan Microeliza Reader (Flow Laboratories, Urvine, UT, USA) at 540 nm. All MTT assays were repeated nine times. The neuroprotective effects of HQ (1 μM), LA (1 μM), and LA-HQ-LA (1 μM) in undifferentiated cells were tested for 24 h in the presence of two neurotoxic agents: H_2_O_2_ (25-150-300 μM) and 6-OHDA (50-75-150 μM).

To obtain a cholinergic phenotype, SH-SY5Y cells were grown in a medium containing retinoic acid (RA) (10 μM) for 3 days; then the medium was removed and replaced with fresh RA (10 μM) medium for other 3 days of differentiation. Alternatively, to have a dopaminergic phenotype, the cells were treated with RA/ phorbol 12-myristate 13-acetate (PMA) in a medium supplemented of RA (10 μM) for 3 days; then the medium was removed and replaced with growth medium containing PMA (80 nM) for a subsequent 3 days. The neuroprotective effects of LA, HQ, and LA-HQ-LA at the concentration of 1 μM were evaluated on differentiated cells exposed to 6-OHDA (25-50-75-150 μM) and H_2_O_2_ (25-150-300 μM).

#### 3.2.2. Measurement of Intracellular ROS

Intracellular ROS were quantified by the 2’,7’-dichlorodihydrofluorescein diacetate (H_2_DCF-DA) assay using a Microplate Fluorometer SPECTRAmax Gemini XS (Molecular Devices, Sunnyvale, CA, U.S.A.) at excitation and emission wavelengths of 480 nm and 530 nm, respectively, and analyzed by SOFTmax Pro software (Version 5.0; Molecular Devices). DCFH-DA is transported across the cell membrane and deacetylated by esterases to form the non-fluorescent 2’,7’-dichlorfluorescein (DCFH). This compound is trapped inside the cells. Next, DCFH is converted to DCF through the action of peroxide rated by the presence of peroxidase. SH-SY5Y cells were plated (2000 cells/well) into special-optics 96-well plates (Corning-Costar). 24 hours later, the cells were washed 3 times with imaging buffer (125 mM NaCl, 5 mM KCl, 1.2 mM MgSO_4_, 5 mM glucose, 25 mM Hepes, 2 mM CaCl_2_). Then 10 μM DCFH-DA media solution were added and the plates were incubated at 37 °C for 30 minutes. After 2 washings with imaging buffer, cells were treated with 25, 150, or 300 μM H_2_O_2_ for immediate fluorescence measurement. The fluorescence intensity is proportional to the ROS levels within the cell cytosol and in inverse proportion to the antioxidant capacity of the cells themselves. Plates, maintained at 25 °C, were read for kinetic analysis in increments from 0 to 5 minutes every 30 seconds [39].

*Statistical analysis*. The statistical analysis (unpaired *t* test) was performed with GraphPad Prism 5 software version 5.0. One-way ANOVA was computed for each level of treatment followed by Dunnett’s *t*-test *post hoc*.

## 4. Conclusions

In conclusion, we synthesized a novel multi-target ligand—containing antioxidant and chelant groups—that might represent a step forward in the search for new molecules against neurodegenerative diseases. LA-HQ-LA was superior to the single molecules (LA and HQ), for the antioxidant and neuroprotective activities against 6-OHDA and H_2_O_2_. Particularly, it displayed a strong neuroprotective effect against all the used concentrations of 6-OHDA, reporting the values similar to those of the control. Clearly, proof of the concept will involve an investigation of its *in vivo* neuroprotective profile.

Although much needs to be understood in terms of pathogenesis of neurodegeneration, it is clear that the simultaneous modulation of oxidative stress and metal brain levels can be considered as a potential strategy to interrupt the vicious cycle that accelerates the progression of the neurodegenerative diseases, such as PD and AD.
